# Innate Immune Effectors in Mycobacterial Infection

**DOI:** 10.1155/2011/347594

**Published:** 2011-01-12

**Authors:** Hiroyuki Saiga, Yosuke Shimada, Kiyoshi Takeda

**Affiliations:** ^1^Laboratory of Immune Regulation, Department of Microbiology and Immunology, Graduate School of Medicine, Osaka University, 2-2, Yamada-oka, Suita, Osaka 565-0871, Japan; ^2^WPI Immunology Frontier Research Center, Osaka University, Suita, Osaka 565-0871, Japan

## Abstract

Tuberculosis, which is caused by infection with *Mycobacterium tuberculosis* (Mtb), remains one of the major bacterial infections worldwide. Host defense against Mtb is mediated by a combination of innate and adaptive immune responses. In the last 15 years, the mechanisms for activation of innate immunity have been elucidated. Toll-like receptors (TLRs) have been revealed to be critical for the recognition of pathogenic microorganisms including mycobacteria. Subsequent studies further revealed that NOD-like receptors and C-type lectin receptors are responsible for the TLR-independent recognition of mycobacteria. Several molecules, such as active vitamin D_3_, secretary leukocyte protease inhibitor, and lipocalin 2, all of which are induced by TLR stimulation, have been shown to direct innate immune responses to mycobacteria. In addition, Irgm1-dependent autophagy has recently been demonstrated to eliminate intracellular mycobacteria. Thus, our understanding of the mechanisms for the innate immune response to mycobacteria is developing.

## 1. Introduction

In humans, tuberculosis is one of deadly infectious diseases. Indeed, approximately 2 million tuberculosis patients die every year. The risk of disease is also increased by emergence of acquired immune deficiency syndrome and development of multidrug-resistant mycobacteria [1]. Therefore, it is important to understand the host defense mechanisms against mycobacteria. Inhalation of aerosols containing *Mycobacterium tuberculosis* (Mtb) causes tuberculosis. After inhalation, Mtb invades alveolar macrophages to enter into the host and establish the infection. The host, in turn, ignites defense responses through sequential activation of immunity, a combination of innate and adaptive immune systems. In the adaptive phase of immune responses, the importance of Th1/IFN-*γ*-mediated responses in mycobacterial infection has been well established [2]. In contrast, although macrophages are the major target of invasion by Mtb, how the innate arm of immunity mediates host defense against mycobacteria had long remained unknown. However, the mechanisms behind innate immune responses have been revealed in the past 15 years following the identification and characterization of pattern recognition receptors (PRRs) such as Toll-like receptors (TLRs) [3]. Furthermore, it has been elucidated that TLR-dependent activation of innate immunity controls the development of adaptive immune responses [4]. The involvement of PRRs other than TLRs in the recognition of mycobacteria has also been revealed. In addition to the induction of adaptive immune responses, the PRR recognition of mycobacteria induces expression of several effector molecules participating in the innate host responses. The role of these innate effector molecules in mycobacterial infection is being elucidated. PRR-independent mechanisms for mycobacterial killing, such as autophagy, have also been revealed. In this paper, we will describe recent advances in our understanding of effectors that mediate innate immune responses against mycobacteria.

## 2. Toll-Like Receptors in Mycobacterial Infection

**Figure 1:**
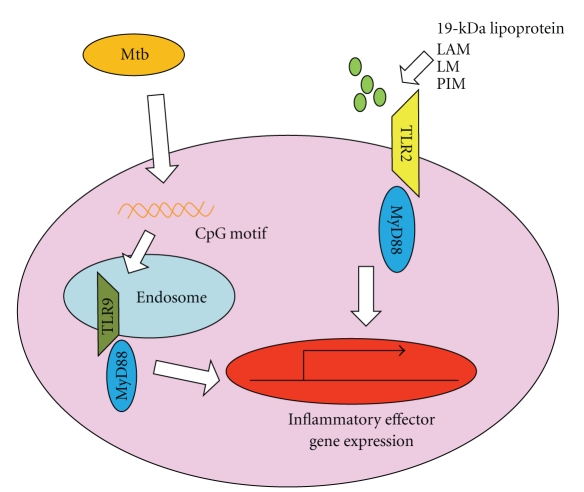
Recognition of mycobacteria by Toll-like receptors. TLR2 recognizes several mycobacterial-derived components. TLR9 recognizes mycobacterial DNA including the CpG motif within endosomal compartments. TLR-dependent recognition of mycobacteria induces activation of signaling pathways via the adaptor molecule MyD88, leading to activation of gene expression.

Innate immune responses after mycobacterial infection are initiated by recognition of mycobacterial components by PRRs, with mycobacterial components activating several TLRs (Figure 1). Genomic DNA from a *Mycobacterium bovis* strain, bacillus Calmette–Guérin (BCG), have an ability to augment NK cell activity and induce type I IFNs from murine spleen cells and human peripheral blood lymphocytes. The immunostimulatory activity of mycobacterial DNA was ascribed to the presence of palindromic sequences including the 5′-CG-3′ motif, now called CpG motif [5], and now known to activate TLR9 [6]. The mycobacterial cell wall consists of several glycolipids. Among these, lipoarabinomannan (LAM) lacking mannose end capping, lipomannan (LM), and phosphatidyl-*myo*-inositol mannoside (PIM) are recognized by TLR2 [7, 8]. The 19-kDa lipoprotein of Mtb also activates macrophages via TLR2 [9, 10]. TLR4 is also presumed to recognize mycobacterial components.

The *in vivo* importance of the TLR-mediated signal in host defense to Mtb was highlighted in studies using mice lacking MyD88, a critical component of TLR signaling. MyD88-deficient mice are highly susceptible to airborne infection with Mtb [11–13]. In contrast to mice lacking MyD88, mice lacking individual TLRs are not dramatically susceptible to Mtb infection. Susceptibility of TLR2-deficient mice to Mtb infection varies between different studies [14, 15], while TLR4-deficient mice do not show high susceptibility to Mtb infection [16, 17]. A report demonstrates that TLR9-deficient mice are susceptible to Mtb infection and mice lacking both TLR2 and TLR9 are more susceptible [18]. These findings indicate that multiple TLRs might be involved in mycobacterial recognition. However, a recent report using mice lacking TLR2/TLR4/TLR9 indicated that these triple KO mice show a milder phenotype than MyD88-deficient mice [12]. Therefore, more intensive examination is required to reveal whether TLRs or molecules other than TLRs activating MyD88 mediate innate immune responses to mycobacterial infection. This study also demonstrated that Th1-like adaptive immune responses are induced even in Mtb-infected MyD88-deficient mice [12]. Therefore, the TLR/MyD88-independent component of innate immunity is involved in the induction of adaptive immune responses during mycobacterial infection. The TLR/MyD88-independent response might be induced by other PRRs described below.

## 3. Non-TLRs in Mycobacterial Infection

**Figure 2:**
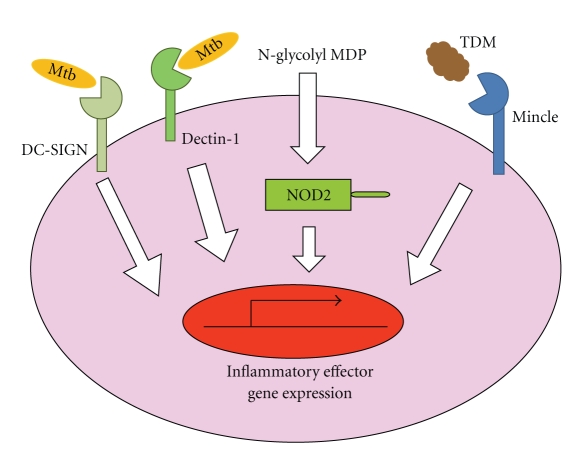
Recognition of mycobacteria by pattern recognition receptors. Several pattern recognition receptors, such as NOD-like receptors and C-type lectin receptors, mediate the TLR-independent recognition of mycobacteria. NOD2, a member of NOD-like receptors, recognizes mycobacterial N-glycolyl MDP within the cytoplasm. DC-SIGN and dectin-1 are members of C-type lectin receptors, which are implicated in the recognition of mycobacteria. In addition, Mincle has been shown to recognize TDM (a mycobacterial cell wall glycolipid).

Several recent findings have indicated that PRRs other than TLRs evoke innate immune responses [19]. These include RIG-I-like receptors, NOD-like receptors (NLRs), and C-type lectin receptors. Among these PRRs, NOD-like receptors and C-type lectin receptors have been implicated in the innate recognition of mycobacteria (Figure 2).

NOD2 is a member of NLRs that recognize muramyl dipeptide (MDP), a core component of bacterial peptidoglycan, in the cytoplasmic compartment. Macrophages from NOD2-deficient mice show a defective cytokine production after Mtb infection [20]. Similarly, mononuclear cells of individuals homozygous for the *3020insC NOD2* mutation show a defective cytokine response after stimulation with Mtb [7]. Activation of the NOD2-mediated pathway is induced by stimulation with live Mtb, but not by heat-killed Mtb [8]. Live Mtb, which is localized in the phagosomal compartment within macrophages, stimulates the cytosolic NOD2 pathway by inducing phagosomal membrane damage [21]. The NOD2 ligand MDP is N-acetylated in most bacteria. However, MDP is N-glycolylated by N-acetyl muramic acid hydroxylase (NamH) in mycobacteria. Analyses using *M. smegmatis* namH mutant and NOD2-deficient mice showed that N-glycolyl MDP is recognized by NOD2. In addition, N-glycolyl MDP is the more potent NOD2 activator than N-acetyl MDP [22]. Thus, NOD2 contributed to the recognition of mycobacteria.

Several members of the NLR family, such as NLRP1, NLRP3, and IPAF, induce assembly of the inflammasome, which leads to caspase-1-dependent secretion of IL-1*β* and IL-18 [23]. The involvement of IL-1*β* and IL-18 in mycobacterial infection was demonstrated in studies using knockout mice [24–27]. A recent study demonstrated that mycobacteria inhibit the inflammasome-dependent casapase-1 activation leading to defective IL-1*β* production [28]. The inhibition of caspase-1 activation has further been shown to be mediated by an Mtb gene, *zmp1*, which encodes a putative Zn^2+^ metalloprotease. Thus, Mtb has a strategy that evades the inflammasome-mediated innate immune responses.

C-type lectin receptors, such as mannose receptor, were originally reported to mediate phagocytosis of mycobacteria [29]. Another C-type lectin receptor, DC-SIGN, has been shown to recognize mycobacteria, and thereby modulate the function of dendritic cells [30–32]. Recognition of mycobacteria by dectin-1 has been shown to induce gene expression such as TNF-*α*, IL-6, and IL-12 [33, 34]. In addition, macrophage inducible C-type lectin (Mincle) has recently been shown to recognize trehalose-6,6′-dimycolate (TDM: also called cord factor), a mycobacterial cell wall glycolipid that is the most studied immunostimulatory component of Mtb [35, 36], thereafter modulating macrophage activation. Thus, several C-type lectin receptors are involved in the recognition of mycobacteria.

CARD9 is involved in the signaling pathways of several PRRs including TLRs, NOD-like receptors, and FcR*γ*-associated C-type lectin receptors through association with Bcl-10 and MALT. Therefore, it is not surprising that CARD9-deficient mice are highly susceptible to Mtb infection. However, interestingly the high susceptibility of CARD9-deficient mice to the infection has been shown to be excessive inflammatory responses due to defective production of the immunosuppressive cytokine IL-10 [37]. Mincle is a member of C-type lectin receptors associated with FcR*γ* [38]. Accordingly, TDM-induced immune responses are mediated by the signaling pathway activating CARD9 [36, 39].

TLRs and C-type lectin receptors are expressed on the plasma membrane or the endosomal/phagosomal membrane, whereas NOD-like receptors are expressed within the cytoplasm. Indeed, distinct patterns of TLR- and NOD-like receptor-mediated gene expression profiles have been demonstrated in infection with intracellular bacteria [40]. Thus, several PRRs recognize mycobacteria in distinct sites within the host cells (macrophages) to synergistically induce effective host defense responses.

## 4. Effectors for Mycobacterial Killing

**Figure 3:**
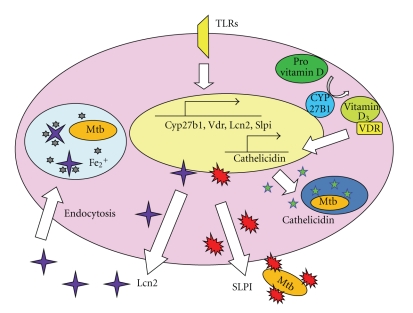
TLR-dependent innate response to mycobacteria. Several TLR-dependent gene products mediate innate immune responses to mycobacteria. Mycobacterial stimulation of TLR2 induces expression of Cyp27b1 and vitamin D receptor (VDR), both of which are involved in vitamin D_3_-dependent induction of cathelicidin which directly kills mycobacteria. TLR-dependent induction of SLPI mediates disruption of the mycobacterial cell wall. Lcn2, which is also induced by TLR stimulation, is internalized into the alveolar epithelial cells and inhibits mycobacterial growth by sequestering iron uptake.

The recognition of mycobacteria by several PRRs induces the expression of several genes that mediate host defense (Figure 3). Among these gene products, vitamin D receptor (VDR) and Cyp27b1, a 25-hydroxyvitamin D_3_ 1-*α*-hydroxylase that catalyzes inactive provitamin D into the bioactive form of vitamin D (1, 25 (OH)_2_D_3_), have been shown to be induced by TLR2 ligands in human macrophages [41]. Stimulation of macrophages with 1, 25 (OH)_2_D_3_ induces the expression of the antimicrobial peptide cathelicidin, and thereby enhances the antimycobacterial killing activity [42]. In addition to cathelicidin, the small cationic antimicrobial peptide defensin mediates innate immune responses to Mtb [43, 44]. Experimental infection of the lung epithelial cell line A549 with Mtb strongly induces production of human *β*-defensin HBD-2, which leads to Mtb killing [43]. HBD-2 expression has also been shown to be induced by TLR2 [45].

Gene expression analyses of the lung of mycobacteria-infected mice have identified several TLR-dependent genes that are involved in innate immune responses during mycobacterial infection. These genes include *Slpi*, encoding secretory leukocyte protease inhibitor (SLPI), and *Lcn2*, encoding lipocalin 2 (Lcn2). SLPI is a secreted protein composed of two cysteine-rich whey acidic protein (WAP) domains [46–48]. SLPI was named after its presence in secretions and its function as a serine protease inhibitor. SLPI was originally shown to mediate wound healing [49, 50]. SLPI is produced by bronchial and alveolar epithelial cells as well as alveolar macrophages and is secreted into the alveolar space at the early phase of mycobacterial respiratory infections. Recombinant mouse SLPI effectively inhibits the *in vitro* growth of BCG and Mtb through disruption of the mycobacterial cell wall structure. Cationic residues within the WAP domains of SLPI are essential for the disruption of mycobacterial cell walls. Moreover, SLPI-deficient mice are highly susceptible to mycobacterial infection [51]. The mechanism by which SLPI attaches to the membrane of mycobacteria has been elucidated. SLPI recognizes mannan-capped lipoarabinomannans and phosphatidylinositol mannoside, which are conserved in mycobacteria. Thus, SLPI might act as a PRR in order to bind to the mycobacterial membrane [52]. 

Lcn2 (also known as neutrophil gelatinase-associated lipocalin, 24p3, or siderocalin) was originally identified in the granules of human neutrophils. Lcn2 is a member of the lipocalin protein family and able to bind to small hydrophobic molecules, siderophore. It is a bacterial molecule made in iron-limited environment and facilitates iron uptake by bacteria [53–58]. The expression of Lcn2 is increased in macrophages of LPS-treated mice [59]. In addition, it is secreted into the alveolar space by alveolar macrophages and epithelial cells during the early phase of respiratory mycobacterial infection. Lcn2 inhibits *in vitro* growth of Mtb by binding the mycobacterial siderophore carboxymycobactin, thereby sequestering iron uptake. Moreover, Lcn2-deficient mice are highly susceptible to intratracheal infection with Mtb. Lcn2 is internalized into alveolar epithelial cells by endocytosis and colocalized with mycobacteria within the cells. Therefore, Lcn2 presumably sequesters iron uptake of mycobacteria within epithelial cells and thereby inhibits their intracellular growth. Within macrophages, the endocytosed Lcn2 and mycobacteria show distinct patterns of subcellular localization, which might allow growth of mycobacteria within macrophages [60]. Thus, Lcn2, which is secreted into the alveolar space during the early phase of mycobacterial infection, is endocytosed into alveolar epithelial cells, thereby inhibiting mycobacterial growth [61].

## 5. Autophagy in Mycobacterial Infection

**Figure 4:**
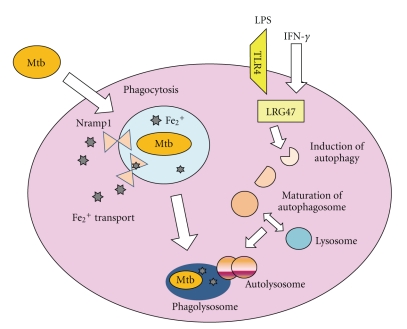
Effectors that mediate mycobacterial killing in macrophages. Macrophages eliminate invading mycobacteria by activating several effector functions, such as phagosomes and autophagy. Nramp1 is expressed in the phagosomal membrane and presumably mediates mycobacterial killing by sequestering iron uptake. IFN-*γ* and the TLR4 ligand induce expression of LRG47, which in turn stimulates autophagy in macrophages. Autophagy is responsible for mycobacterial killing by promoting fusion of mycobacterial phagosomes to lysosomes.

Phagocytosis of myobacteria and PRR-dependent recognition of mycobacteria activate several effector functions in macrophages (Figure 4). Maturation of phagosomes is a crucial step in the elimination of intracellular bacteria. The natural-resistance-associated macrophage protein (Nramp1), which is encoded by Slc11a1, is thought to mediate transportation of divalent cations in the phagosomal membrane and thereby sequesters iron (Fe^2+^) from mycobacteria to enhance bacterial killing by macrophages [62]. Polymorphisms of the *SLC11A1 *gene have been associated with susceptibility to several infectious diseases, including tuberculosis [63, 64]. However, *in vivo* studies have shown that Nramp1-deficient mice are not more susceptible than wild-type mice to infection with virulent Mtb [65]. Thus, the role of Nramp1 in mycobacterial infection is still controversial. This might be due to the presence of other killing mechanisms for mycobacteria in macrophages. Indeed, autophagy has recently been shown to be involved in host defense against several intracellular pathogens that reside within phagosomes [66]. Autophagy was originally identified as a homeostatic mechanism for the catabolic reaction of cellular constitutes [67, 68]. It has been demonstrated that autophagy mediates innate immune responses against mycobacteria by promoting phagolysosomal maturation within macrophages [69, 70]. Autophagy is induced by IFN-*γ*-dependent induction of a member of the immunity-related p47 guanosine triphosphatases (IRG) family, LRG47 (also known as Irgm1) in murine macrophages [69]. The importance of LRG47 in resistance to Mtb infection was demonstrated in LRG47-deficient mice, which show high susceptibility to infection [71]. A subsequent study demonstrated that stimulation of macrophages with the TLR4 ligand LPS leads to the MyD88-independent induction of autophagy, which enhances mycobacterial colocalization with the autophagosomes. Since LPS stimulation induces expression of LRG47, the TLR signaling establishes a close relationship between innate immunity and autophagy in mycobacterial infection [72]. In humans, the most equivalent gene to murine Irgm1 is IRGM. IRGM has also been implicated in the induction of autophagy in mycobacteria-infected human macrophages [73]. Irgm1 has been shown to associate with the mycobacterial phagosome by interacting with phosphatidylinositol-3,4-bisphosphate (PtdIns(3,4)P(2)) and PtdIns(3,4,5)P(3) [74]. The connection of the IRG family of proteins with autophagy has been further demonstrated in an alternative intracellular infection model. In this study, Irgm3 (also known as IGTP) has been implicated in autophagy induction in macrophages infected with *Toxoplasma gondii *[75].

p62 (also called A170 or SQSTM1) directly binds to cytosolic polyubiquitinated proteins and thereby induces their autophagic clearance [76, 77]. It has also been shown that p62 targets intracellular *Salmonella typhimurium* decorated by ubiquitinated proteins to induce autophagy [78]. In the case of mycobacteria residing in the phagosome, p62 delivers cytosolic ubiquitinated proteins to autophagolysosomes where they are proteolytically processed to products that are able to kill mycobacteria [79]. In accordance with this finding, it has been shown that mycobacterial killing by ubiquitin-derived peptides is enhanced by autophagy [80]. 

As described above, 1, 25 (OH)_2_D_3_ mediates antimycobacterial activity via induction of cathelicidin. A recent report demonstrated that 1, 25 (OH)_2_D_3_-mediated expression of cathelicidin induces autophagy [81]. Thus, several innate immune effectors are closely interacted.

## 6. Human Genetics in Tuberculosis

In addition to the intensive studies using murine models, considerable advances have been made in our understanding of the susceptibility to Mtb infection in humans through the identification of mutations and polymorphisms of innate immunity-related genes in tuberculosis patients. As described above, polymorphisms of the *SLC11A1 *gene are associated with tuberculosis. Subsequent studies identified a significant distinction between tuberculosis patients and healthy controls in *TLR2* Arg753Gln polymorphism genotype, indicating that the *TLR2* polymorphism influences the susceptibility of Mtb infection [82]. *VDR *polymorphisms have also been implicated in the susceptibility of Mtb infection [83]. These studies suggest that several genes, which have been revealed to be critical in innate responses in mouse models of Mtb infection, regulate Mtb infection in humans.

## 7. Conclusion

Since the discovery of TLRs at the end of the 20th century, rapid advances have been made in our understanding of the mechanisms for activation of innate immunity. Accordingly, innate immunity has been revealed to have a pivotal role in host defense against mycobacteria. The TLR-independent mechanisms for the innate immune response to mycobacteria have also been elucidated. The emergence of multidrug-resistant Mtb is now a major public health problem all over the world. In this context, it is highly critical to develop a new strategy for the treatment of Mtb-infected patients that supplements the conventional antimycobacterial chemotherapeutic drugs. More precise understanding of the innate immune response to Mtb will pave the way for the development of an effective drug that targets the host innate immunity for the treatment of tuberculosis.

## References

[1] Kaufmann SHE, McMichael AJ (2005). Annulling a dangerous liaison: vaccination strategies against AIDS and tuberculosis. *Nature Medicine*.

[2] Kaufmann SHE (2001). How can immunology contribute to the control of tuberculosis?. *Nature Reviews Immunology*.

[3] Janeway CA, Medzhitov R (2002). Innate immune recognition. *Annual Review of Immunology*.

[4] Akira S, Takeda K, Kaisho T (2001). Toll-like receptors: critical proteins linking innate and acquired immunity. *Nature Immunology*.

[5] Kuramoto E, Yano O, Kimura Y (1992). Oligonucleotide sequences required for natural killer cell activation. *Japanese Journal of Cancer Research*.

[6] Hemmi H, Takeuchi O, Kawai T (2000). A Toll-like receptor recognizes bacterial DNA. *Nature*.

[7] Ferwerda G, Girardin SE, Kullberg BJ (2005). NOD2 and toll-like receptors are nonredundant recognition systems of Mycobacterium tuberculosis. *PLoS Pathogens*.

[8] Yang Y, Yin C, Pandey A, Abbott D, Sassetti C, Kelliher MA (2007). NOD2 pathway activation by MDP or Mycobacterium tuberculosis infection involves the stable polyubiquitination of Rip2. *Journal of Biological Chemistry*.

[9] Aliprantis AO, Yang RB, Mark MR (1999). Cell activation and apoptosis by bacterial lipoproteins through Toll- like receptor-2. *Science*.

[10] Brightbill HD, Libraty DH, Krutzik SR (1999). Host defense mechanisms triggered by microbial lipoproteins through toll-like receptors. *Science*.

[11] Fremond CM, Yeremeev V, Nicolle DM, Jacobs M, Quesniaux VF, Ryffel B (2004). Fatal Mycobacterium tuberculosis infection despite adaptive immune response in the absence of MyD88. *Journal of Clinical Investigation*.

[12] Hölscher C, Reiling N, Schaible UE (2008). Containment of aerogenic Mycobacterium tuberculosis infection in mice does not require MyD88 adaptor function for TLR2, -4 and -9. *European Journal of Immunology*.

[13] Scanga CA, Bafica A, Feng CG, Cheever AW, Hieny S, Sher A (2004). MyD88-deficient mice display a profound loss in resistance to Mycobacterium tuberculosis associated with partially impaired Th1 cytokine and nitric oxide synthase 2 expression. *Infection and Immunity*.

[14] Drennan MB, Nicolle D, Quesniaux VJF (2004). Toll-like receptor 2-deficient mice succumb to Mycobacterium tuberculosis infection. *American Journal of Pathology*.

[15] Sugawara I, Yamada H, Li C, Mizuno S, Takeuchi O, Akira S (2003). Mycobacterial infection in TLR2 and TLR6 knockout mice. *Microbiology and Immunology*.

[16] Heldwein KA, Liang MD, Andresen TK (2003). TLR2 and TLR4 serve distinct roles in the host immune response against Mycobacterium bovis BCG. *Journal of Leukocyte Biology*.

[17] Reiling N, Hölscher C, Fehrenbach A (2002). Cutting edge: Toll-like receptor (TLR)2- and TLR4-mediated pathogen recognition in resistance to airborne infection with Mycobacterium tuberculosis. *Journal of Immunology*.

[18] Bafica A, Scanga CA, Feng CG, Leifer C, Cheever A, Sher A (2005). TLR9 regulates Th1 responses and cooperates with TLR2 in mediating optimal resistance to Mycobacterium tuberculosis. *Journal of Experimental Medicine*.

[19] Takeuchi O, Akira S (2010). Pattern recognition receptors and inflammation. *Cell*.

[20] Divangahi M, Mostowy S, Coulombe F (2008). NOD2-deficient mice have impaired resistance to Mycobacterium tuberculosis infection through defective innate and adaptive immunity. *Journal of Immunology*.

[21] Pandey AK, Yang Y, Jiang Z (2009). Nod2, Rip2 and Irf5 play a critical role in the type I interferon response to Mycobacterium tuberculosis. *PLoS Pathogens*.

[22] Coulombe F, Divangahi M, Veyrier F (2009). Increased NOD2-mediated recognition of N-glycolyl muramyl dipeptide. *Journal of Experimental Medicine*.

[23] Schroder K, Tschopp J (2010). The Inflammasomes. *Cell*.

[24] Yamada H, Mizumo S, Horai R, Iwakura Y, Sugawara I (2000). Protective role of interleukin-1 in mycobacterial infection in IL-1 *α*/*β* double-knockout mice. *Laboratory Investigation*.

[25] Juffermans NP, Florquin S, Camoglio L (2000). Interleukin-1 signaling is essential for host defense during murine pulmonary tuberculosis. *Journal of Infectious Diseases*.

[26] Fremond CM, Togbe D, Doz E (2007). IL-1 receptor-mediated signal is an essential component of MyD88-dependent innate response to Mycobacterium tuberculosis infection. *Journal of Immunology*.

[27] Sugawara I, Yamada H, Kaneko H, Mizuno S, Takeda K, Akira S (1999). Role of interleukin-18 (IL-18) in mycobacterial infection in IL-18- gene-disrupted mice. *Infection and Immunity*.

[28] Master SS, Rampini SK, Davis AS (2008). Mycobacterium tuberculosis prevents inflammasome activation. *Cell Host and Microbe*.

[29] Kang PB, Azad AK, Torrelles JB (2005). The human macrophage mannose receptor directs Mycobacterium tuberculosis lipoarabinomannan-mediated phagosome biogenesis. *Journal of Experimental Medicine*.

[30] Tailleux L, Neyrolles O, Honoré-Bouakline S (2003). Constrained intracellular survival of Mycobacterium tuberculosis in human dendritic cells. *Journal of Immunology*.

[31] Geijtenbeek TBH, Van Vliet SJ, Koppel EA (2003). Mycobacteria target DC-SIGN to suppress dendritic cell function. *Journal of Experimental Medicine*.

[32] Gringhuis SI, den Dunnen J, Litjens M, van der Vlist M, Geijtenbeek TBH (2009). Carbohydrate-specific signaling through the DC-SIGN signalosome tailors immunity to Mycobacterium tuberculosis, HIV-1 and Helicobacter pylori. *Nature Immunology*.

[33] Yadav M, Schorey JS (2006). The *β*-glucan receptor dectin-1 functions together with TLR2 to mediate macrophage activation by mycobacteria. *Blood*.

[34] Rothfuchs AG, Bafica A, Feng CG (2007). Dectin-1 interaction with Mycobacterium tuberculosis leads to enhanced IL-12p40 production by splenic dendritic cells. *Journal of Immunology*.

[35] Ishikawa E, Ishikawa T, Morita YS (2009). Direct recognition of the mycobacterial glycolipid, trehalose dimycolate, by C-type lectin Mincle. *Journal of Experimental Medicine*.

[36] Schoenen H, Bodendorfer B, Hitchens K (2010). Cutting Edge: Mincle is essential for recognition and adjuvanticity of the mycobacterial cord factor and its synthetic analog trehalose-dibehenate. *Journal of Immunology*.

[37] Dorhoi A, Desel C, Yeremeev V (2010). The adaptor molecule CARD9 is essential for tuberculosis control. *Journal of Experimental Medicine*.

[38] Yamasaki S, Ishikawa E, Sakuma M, Hara H, Ogata K, Saito T (2008). Mincle is an ITAM-coupled activating receptor that senses damaged cells. *Nature Immunology*.

[39] Werninghaus K, Babiak A, Groß O (2009). Adjuvanticity of a synthetic cord factor analogue for subunit Mycobacterium tuberculosis vaccination requires FclR*γ*-Syk- Card9-dependent innate immune activation. *Journal of Experimental Medicine*.

[40] Leber JH, Crimmins GT, Raghavan S, Meyer-Morse NP, Cox JS, Portnoy DA (2008). Distinct TLR- and NLR-mediated transcriptional responses to an intracellular pathogen.. *PLoS Pathogens*.

[41] Liu PT, Stenger S, Li H (2006). Toll-like receptor triggering of a vitamin D-mediated human antimicrobial response. *Science*.

[42] Liu PT, Stenger S, Tang DH, Modlin RL (2007). Cutting edge: vitamin D-mediated human antimicrobial activity against Mycobacterium tuberculosis is dependent on the induction of cathelicidin. *Journal of Immunology*.

[43] Rivas-Santiago B, Schwander SK, Sarabia C (2005). Human *β*-defensin 2 is expressed and associated with Mycobacterium tuberculosis during infection of human alveolar epithelial cells. *Infection and Immunity*.

[44] Rivas-Santiago B, Contreras JCL, Sada E, Hernández-Pando R (2008). The potential role of lung epithelial cells and *β*-defensins in experimental latent tuberculosis. *Scandinavian Journal of Immunology*.

[45] Kumar A, Zhang J, Yu FSX (2006). Toll-like receptor 2-mediated expression of *β*-defensin-2 in human corneal epithelial cells. *Microbes and Infection*.

[46] Clauss A, Lilja H, Lundwall Å (2005). The evolution of a genetic locus encoding small serine proteinase inhibitors. *Biochemical and Biophysical Research Communications*.

[47] Eisenberg SP, Hale KK, Heimdal P, Thompson RC (1990). Location of the protease-inhibitory region of secretory leukocyte protease inhibitor. *Journal of Biological Chemistry*.

[48] Hagiwara K, Kikuchi T, Endo Y (2003). Mouse SWAM1 and SWAM2 are antibacterial proteins composed of a single whey acidic protein motif. *Journal of Immunology*.

[49] Ashcroft GS, Lei K, Jin W (2000). Secretory leukocyte protease inhibitor mediates non-redundant functions necessary for normal wound healing. *Nature Medicine*.

[50] Zhu J, Nathan C, Jin W (2002). Conversion of proepithelin to epithelins: roles of SLPI and elastase in host defense and wound repair. *Cell*.

[51] Nishimura J, Saiga H, Sato S (2008). Potent antimycobacterial activity of mouse secretory leukocyte protease inhibitor. *Journal of Immunology*.

[52] Gomez SA, Arguelles CL, Guerrieri D (2009). Secretory leukocyte protease inhibitor a secreted pattern recognition receptor for mycobacteria. *American Journal of Respiratory and Critical Care Medicine*.

[53] Kjeldsen L, Cowland JB, Borregaard N (2000). Human neutrophil gelatinase-associated lipocalin and homologous proteins in rat and mouse. *Biochimica et Biophysica Acta*.

[54] Devireddy LR, Teodoro JG, Richard FA, Green MR (2001). Induction of apoptosis by a secreted lipocalin that is transcriptionally regulated by IL-3 deprivation. *Science*.

[55] Kjeldsen L, Johnsen AH, Sengelov H, Borregaard N (1993). Isolation and primary structure of NGAL, a novel protein associated with human neutrophil gelatinase. *Journal of Biological Chemistry*.

[56] Flower DR, North ACT, Attwood TK (1991). Mouse oncogene protein 24p3 is a member of the lipocalin protein family. *Biochemical and Biophysical Research Communications*.

[57] Liu Q, Ryon J, Nilsen-Hamilton M (1997). Uterocalin: a mouse acute phase protein expressed in the uterus around birth. *Molecular Reproduction and Development*.

[58] Goetz DH, Holmes MA, Borregaard N, Bluhm ME, Raymond KN, Strong RK (2002). The neutrophil lipocalin NGAL is a bacteriostatic agent that interferes with siderophore-mediated iron acquisition. *Molecular Cell*.

[59] Flo TH, Smith KD, Sato S (2004). Lipocalin 2 mediates an innate immune response to bacterial infection by sequestrating iron. *Nature*.

[60] Halaas Ø, Steigedal M, Haug M (2010). Intracellular mycobacterium avium intersect transferrin in the Rab11M^+^ recycling endocytic pathway and avoid lipocalin 2 trafficking to the lysosomal pathway. *Journal of Infectious Diseases*.

[61] Saiga H, Nishimura J, Kuwata H (2008). Lipocalin 2-dependent inhibition of mycobacterial growth in alveolar epithelium. *Journal of Immunology*.

[62] Jabado N, Jankowski A, Dougaparsad S, Picard V, Grinstein S, Gros P (2000). Natural resistance to intracellular infections: natural resistance-associated macrophage protein 1 (NRAMP1) functions as a pH-dependent manganese transporter at the phagosomal membrane. *Journal of Experimental Medicine*.

[63] Bellamy R, Ruwende C, Corrah T, McAdam KPWJ, Whittle HC, Hill AVS (1998). Variations in the NRAMP1 gene and susceptibility to tuberculosis in West Africans. *New England Journal of Medicine*.

[64] Malik S, Abel L, Tooker H (2005). Alleles of the NRAMP1 gene are risk factors for pediatric tuberculosis disease. *Proceedings of the National Academy of Sciences of the United States of America*.

[65] North RJ, LaCourse R, Ryan L, Gros P (1999). Consequence of Nramp1 deletion to Mycobacterium tuberculosis infection in mice. *Infection and Immunity*.

[66] Deretic V (2009). Multiple regulatory and effector roles of autophagy in immunity. *Current Opinion in Immunology*.

[67] Kuma A, Hatano M, Matsui M (2004). The role of autophagy during the early neonatal starvation period. *Nature*.

[68] Shintani T, Klionsky DJ (2004). Autophagy in health and disease: a double-edged sword. *Science*.

[69] Gutierrez MG, Master SS, Singh SB, Taylor GA, Colombo MI, Deretic V (2004). Autophagy is a defense mechanism inhibiting BCG and Mycobacterium tuberculosis survival in infected macrophages. *Cell*.

[70] Delgado MA, Elmaoued RA, Davis AS, Kyei G, Deretic V (2008). Toll-like receptors control autophagy. *EMBO Journal*.

[71] Feng CG, Collazo-Custodio CM, Eckhaus M (2004). Mice deficient in LRG-47 display increased susceptibility to mycobacterial infection associated with the induction of lymphopenia. *Journal of Immunology*.

[72] Xu YI, Jagannath C, Liu XD, Sharafkhaneh A, Kolodziejska KE, Eissa NT (2007). Toll-like receptor 4 is a sensor for autophagy associated with innate immunity. *Immunity*.

[73] Singh SB, Davis AS, Taylor GA, Deretic V (2006). Human IRGM induces autophagy to eliminate intracellular mycobacteria. *Science*.

[74] Tiwari S, Choi HP, Matsuzawa T, Pypaert M, MacMicking JD (2009). Targeting of the GTPase Irgm1 to the phagosomal membrane via PtdIns(3,4)P_2_ and PtdIns(3,4,5)P_3_ promotes immunity to mycobacteria. *Nature Immunology*.

[75] Ling YM, Shaw MH, Ayala C (2006). Vacuolar and plasma membrane stripping and autophagic elimination of Toxoplasma gondii in primed effector macrophages. *Journal of Experimental Medicine*.

[76] Bjørkøy G, Lamark T, Brech A (2005). p62/SQSTM1 forms protein aggregates degraded by autophagy and has a protective effect on huntingtin-induced cell death. *Journal of Cell Biology*.

[77] Pankiv S, Clausen TH, Lamark T (2007). p62/SQSTM1 binds directly to Atg8/LC3 to facilitate degradation of ubiquitinated protein aggregates by autophagy. *Journal of Biological Chemistry*.

[78] Zheng YT, Shahnazari S, Brech A, Lamark T, Johansen T, Brumell JH (2009). The adaptor protein p62/SQSTM1 targets invading bacteria to the autophagy pathway. *Journal of Immunology*.

[79] Ponpuak M, Davis AS, Roberts EA (2010). Delivery of cytosolic components by autophagic adaptor protein p62 endows autophagosomes with unique antimicrobial properties. *Immunity*.

[80] Alonso S, Pethe K, Russell DG, Purdy GE (2007). Lysosomal killing of Mycobacterium mediated by ubiquitin-derived peptides is enhanced by autophagy. *Proceedings of the National Academy of Sciences of the United States of America*.

[81] Yuk JM, Shin DM, Lee HM (2009). Vitamin D3 induces autophagy in human monocytes/macrophages via cathelicidin. *Cell Host and Microbe*.

[82] Ogus AC, Yoldas B, Ozdemir T (2004). The Arg753Gln polymorphism of the human Toll-like receptor 2 gene in tuberculosis disease. *European Respiratory Journal*.

[83] Uitterlinden AG, Fang Y, Van Meurs JBJ, Pols HAP, Van Leeuwen JPTM (2004). Genetics and biology of vitamin D receptor polymorphisms. *Gene*.

